# Chloroplast phylogenomic insights into the evolution of *Distylium* (Hamamelidaceae)

**DOI:** 10.1186/s12864-021-07590-6

**Published:** 2021-04-22

**Authors:** Wenpan Dong, Yanlei Liu, Chao Xu, Yongwei Gao, Qingjun Yuan, Zhili Suo, Zhixiang Zhang, Jiahui Sun

**Affiliations:** 1 Laboratory of Systematic Evolution and Biogeography of Woody Plants, School of Ecology and Nature Conservation, Beijing Forestry University, Beijing, 100083 China; 2 State Key Laboratory of Systematic and Evolutionary Botany, Institute of Botany, Chinese Academy of Sciences, Beijing, 100093 China; 3 State Key Laboratory Breeding Base of Dao-di Herbs, National Resource Center for Chinese Materia Medica, China Academy of Chinese Medical Sciences, Beijing, 100700 China

**Keywords:** *Distylium*, Hamamelidaceae, Species identification, cpDNA marker, Phylogenomics

## Abstract

**Background:**

Most *Distylium* species are endangered. *Distylium* species mostly display homoplasy in their flowers and fruits, and are classified primarily based on leaf morphology. However, leaf size, shape, and serration vary tremendously making it difficult to use those characters to identify most species and a significant challenge to address the taxonomy of *Distylium.* To infer robust relationships and develop variable markers to identify *Distylium* species, we sequenced most of the *Distylium* species chloroplast genomes.

**Results:**

The *Distylium* chloroplast genome size was 159,041–159,127 bp and encoded 80 protein-coding, 30 transfer RNAs, and 4 ribosomal RNA genes. There was a conserved gene order and a typical quadripartite structure. Phylogenomic analysis based on whole chloroplast genome sequences yielded a highly resolved phylogenetic tree and formed a monophyletic group containing four *Distylium* clades. A dating analysis suggested that *Distylium* originated in the Oligocene (34.39 Ma) and diversified within approximately 1 Ma. The evidence shows that *Distylium* is a rapidly radiating group. Four highly variable markers, *matK-trnK*, *ndhC-trnV*, *ycf1*, and *trnT-trnL*, and 74 polymorphic simple sequence repeats were discovered in the *Distylium* plastomes.

**Conclusions:**

The plastome sequences had sufficient polymorphic information to resolve phylogenetic relationships and identify *Distylium* species accurately.

**Supplementary Information:**

The online version contains supplementary material available at 10.1186/s12864-021-07590-6.

## Background

*Distylium* Sieb. et Zucc is a genus of flowering plants in the tribe Fothergilleae of the family Hamamelidaceae, which is endemic to Asia. Fifteen species have been reported in *Distylium* worldwide, with 12 species occurring in China (*D. chinense*, 2n = 24). Additionally, two species are found in Japan, one of which is found also in China, and one species each in Malaysia and India. They are evergreen shrubs or small trees that grow mostly in subtropical evergreen forests.

This genus has been introduced as a cultivar and thrives in warm temperate and subtropical climates in Europe and the United States. *Distylium*, with dense branches and deep evergreen leaves, a neat tree shape, small red flowers in spring, good soundproof effects, and strong resistance to smoke and dust and various toxic gases (e.g., sulfur dioxide and chlorine), are suitable as greening and ornamental plants in cities, and industrial and mining areas. They are commonly cultivated in urban gardens in the Yangtze River basin of China. Some species, such as *D. chinense,* are used to stabilize solid earth embankments because of their robust root system, flooding tolerance, and resistance to sand burial soaks [1, 2].

Most *Distylium* species are endangered. According to the threatened species list of China’s higher plants [3], two species are Critically Endangered species (*D. macrophyllum* and *D. tsiangii*), two are Endangered species (*D. chinense* and *D. gracile*), and two species are Vulnerable (*D. chungii* and *D. elaeagnoides*). Some *Distylium* species are narrowly distributed, such as *D. lepidotum*, which is endemic to the Ogasawara (Bonin) Islands, located in the northwestern Pacific approximately 1000 km south of Tokyo [4]. *D. tsiangii* is only located in Dushan and Bazai counties of Guizhou Province.

*Distylium* species lack significant differences in the morphology of their flowers and fruits, and are classified primarily based on leaf morphology. However, leaf size, shape, and serration vary tremendously and are difficult characters to use in most cases. For example, the range of leaf variation in *D. buxifolium* is very striking [5]. This variability has led to a proposed number of new species, which have been reduced to synonymy, as more material has been found to link extreme forms [5]. Due to the insufficient number of morphological diagnostic characters and highly polymorphic traits, taxonomic classification of *Distylium* species has been unclear. Chloroplast genome markers, such as *atpB*, *atpB-rbcL*, *matK*, *rbcL*, *trnH-psbA*, and *trnL-F*, and the internal transcribed spacer (ITS) has enabled molecular phylogenetic analyses of several *Distylium* species [6–9]. However, those markers have lower divergence among *Distylium* species; no study has inferred the phylogeny of this genus.

Whole chloroplast genome sequences have been widely used to infer phylogenetic relationships at different taxonomic levels, and provide an effective genetic resource for resolving complex evolutionary relationships and identifying ambiguous species. With the development of sequencing methods, complete chloroplast genome sequences are now available at low cost, extending gene-based phylogenetics to genome-based phylogenomics [10–12], extending gene-based species identification to genome-based super DNA barcoding [13, 14], and making it easier to study evolutionary events in plant species [15].

In this study, we specifically aimed to (1) develop and screen appropriate intrageneric markers in the chloroplast genome to establish DNA barcodes for *Distylium*; (2) estimate the effectiveness of a whole chloroplast genome data set in resolving the relationships within this radiating lineage; (3) estimate the divergence time of *Distylium*.

## Results

### Basic characteristics of the *Distylium* plastomes

**Fig. 1:**
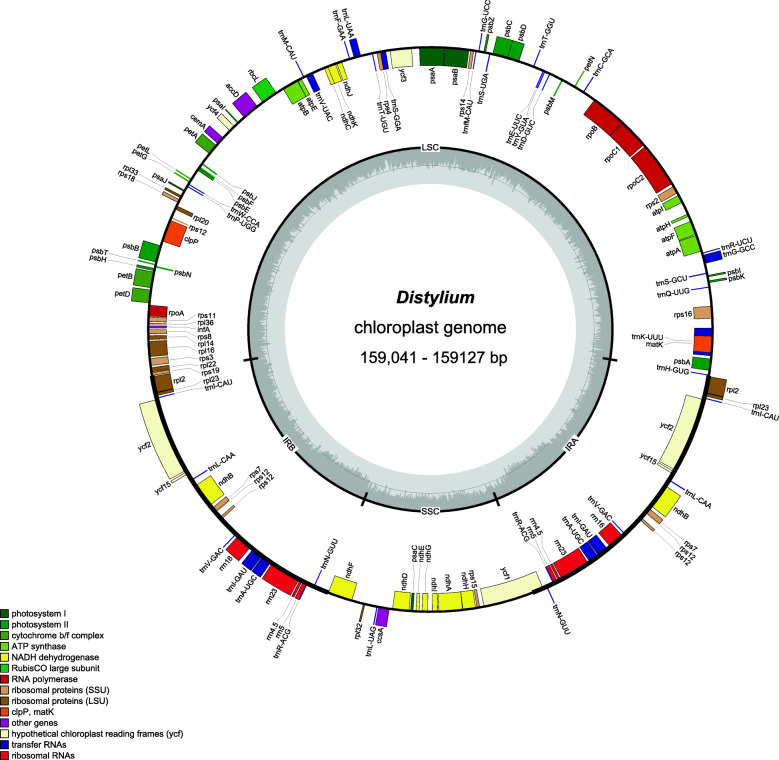
Gene map of the *Distylium* plastomes. Genes shown inside the inner circle are transcribed counterclockwise and those outside the circle are transcribed clockwise. The GC content of the genome is indicated by the dashed area in the inner circle

The complete chloroplast genomes of the 12 newly sequenced *Distylium* species ranged in length from 159,041 bp (*D. lepidoium*) to 159,127 bp (*D. gracile*) (Table 1). The *Distylium* chloroplast genomes had a quadripartite structure typical of most angiosperm species, including large single copy (LSC) and small single copy (SSC) regions separated by two inverted repeat (IRa and IRb) regions (Fig. 1). The LSC regions ranged from 87,825 bp (*D. pingpienense*) to 87,863 bp (*D. racemosum*), the SSC regions varied between 18,770 bp (*D. dunnianum*) and 18,796 bp (*D. lepidoium*), and the IR regions ranged from 26,225 bp (*D. elaeagnoides*) to 26,241 bp (*D. dunnianum*). The GC content of the chloroplast genome sequences was 38.0%. A total of 114 unique genes was detected in the chloroplast genomes of the 11 *Distylium* species (Table S1), including 80 protein coding genes, 30 tRNA genes, and 4 rRNA genes, and the gene order was highly conserved (Fig. 1 and Table 1). A total of 18 genes (including 11 coding genes and seven tRNA genes) had introns, with 16 genes having one intron and two genes (*ycf3* and *clpP*) having two introns in the *Distylium* chloroplast genomes.

**Table 1:** The basic plastomes information of 12 *Distylium* samples

Species	Nucleatide length (bp)				Nmuber of genes			GC%				Genbank accession number
	Total	LSC	SSC	IR	Protein	tRNA	rRNA	Total	LSC	SSC	IR	
*D. buxifolium*	159,084	87,828	18,790	26,233	80	30	4	38.0	36.2	32.5	43.1	MW248115
*D. chinese*	159,087	87,830	18,791	26,233	80	30	4	38.0	36.2	32.5	43.1	MW248112
*D. cuspidatum*	159,068	87,848	18,784	26,218	80	30	4	38.0	36.2	32.4	43.1	MW248117
*D. dunnianum*	159,097	87,845	18,770	26,241	80	30	4	38.0	36.2	32.5	43.1	MW248109
*D. elaeagnoides*	159,094	87,857	18,787	26,225	80	30	4	38.0	36.2	32.5	43.1	MW248120
*D. gracile*	159,127	87,854	18,793	26,240	80	30	4	38.0	36.2	32.5	43.0	MW248116
*D. lepidoium*	159,041	87,831	18,796	26,205	80	30	4	38.0	36.2	32.5	43.1	MW248118
*D. lepidoium*	159,042	87,832	18,796	26,205	80	30	4	38.0	36.2	32.5	43.1	MW248119
*D. macrophyllum*	159,095	87,847	18,788	26,230	80	30	4	38.0	36.2	32.5	43.1	MW248111
*D. myricoides*	159,093	87,847	18,780	26,233	80	30	4	38.0	36.2	32.5	43.1	MW248110
*D. pingpienense*	159,081	87,825	18,790	26,233	80	30	4	38.0	36.2	32.5	43.1	MW248114
*D. racemosum*	159,107	87,863	18,782	26,231	80	30	4	38.0	36.2	32.5	43.1	MW248113

### Repetitive sequences

**Fig. 2:**
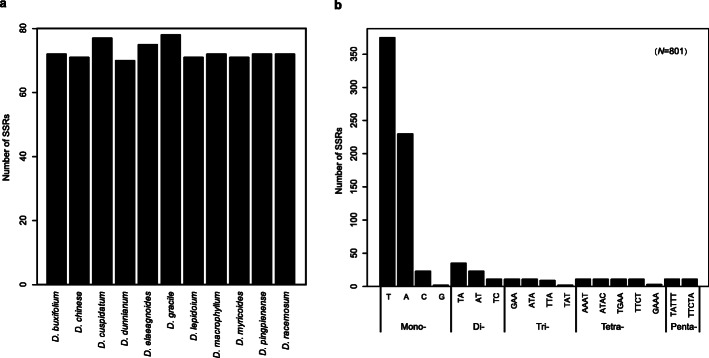
Frequency of the simple sequence repeat (SSR) sequences in the *Distylium* plastomes. **a.** The number of SSRs detected in the 11 *Distylium* species; **b**. Frequency of SSRs with di- to penta-nucleotide motifs

A total of 801 SSRs were identified across the chloroplast genomes of the 11 *Distylium* species (Fig. 2 and Table S2). The number of SSRs per species ranged from 70 (*D. dunnianum*) to 78 (*D. gracile*). The majority of the SSRs were mononucleotide repeats (78.65%), followed by dinucleotide (8.61%) and tetranucleotide (5.87%) repeats. There were no hexanucleotide repeats in the *Distylium* plastomes. The SSR A and T motifs were the most frequent. SSRs were particularly rich in AT in the *Distylium* plastomes. Among those SSRs, most were located in the LSC/SSC regions (94.01%).

A total of 96 unique SSRs and 74 SSRs were polymorphic across the 11 *Distylium* species. All polymorphic SSRs were located in the single copy regions, except two SSRs (Table 2). The mononucleotide repeat units A and T were also the most frequent polymorphic SSRs.

**Table 2:** Polymorphism of SSRs in *Distylium* plastomes

Regions/SSR unit	Overall	Polymorphic	Monomorphic
LSC	80	60	20
IR	2	2	0
SSC	14	12	2
A	30	30	0
T	42	36	6
C	3	0	3
G	1	1	0
TA	4	2	2
AT	3	1	2
TC	1	0	1
TTA	2	1	1
ATA	1	0	1
TAT	1	1	0
GAA	1	0	1
AAAT	1	0	1
ATAC	1	0	1
GAAA	1	1	0
TATTT	1	0	1
TGAA	1	0	1
TTCT	1	1	0
TTCTA	1	0	1
Total	96	74	22

### Indel variations

**Fig. 3:**
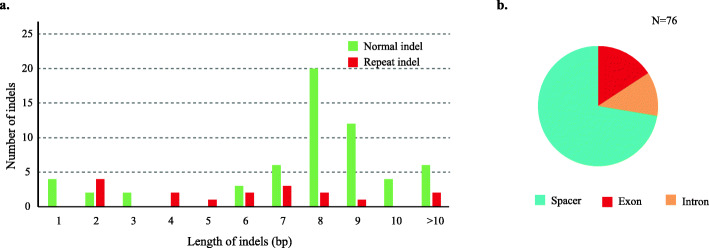
Analyses of indels in the *Distylium* plastomes. **a**. Number and size of the indels among the *Distylium* plastomes. **b**. Frequency of indel types and locations

A total of 76 indels were discovered in the *Distylium* plastomes, including 59 normal indels and 17 repeat indels. Most of the indels (72.37%, 55 times) were located in the spacer regions, 15.79% (12 times) of indels occurred in the exons, and 11.84% (nine times) were found in the introns (Fig. 3). The *trnT-trnL* spacer had five indels, followed by *ndhC-trnV* (3 indels). The size of the normal indels ranged from 1 to 13 bp, with 8 bp and 9 bp length indels being the most common. The largest indel (13 bp) was located in the *trnC-petN* spacer and was a deletion in *D. macrophyllum*. The second largest indel was in the *ycf1* exon of 12 bp length and was an insert in the two *D. lepidoium* samples. The length of the repeat indels ranged from 2 to 16 bp. The largest repeat indel occurred in the *rpl20-rps12* spacer and the second largest repeat indel was located in the *rps7-trnV* spacer.

### Variation in the plastomes and molecular markers for *Distylium* species

The mVISTA results showed that the 11 *Distylium* chloroplast genomes were collinear and highly conserved (Figure S1). The entire chloroplast genome of the 11 *Distylium* species was 159,360 bp in length, including 298 polymorphic sites and 115 parsimony informative sites (Table 3). The overall nucleotide diversity (π) was 0.00045; however, each region of the chloroplast genome revealed different nucleotide diversity; the SSC exhibited the highest π value (0.00089) and the IR had the lowest π value (0.00006). All species had a unique chloroplast haplotype. The number of nucleotide substitutions among the 11 species varied from 7 to 109, and the p-distance varied from 0.0004 to 0.0069. The lowest divergence was observed between *D. buxifolium* and *D. chinese*, and the largest sequence divergence was observed between *D. chinese* and *D. lepidoium*.

**Table 3:** Sequences divergence of *Distylium* plastomes

Regions	Alignment length (bp)	Number of variable sites			Nucleotide polymorphism	
		Polymorphic	Singleton	Parsimony informative	Nucleotide diversity	Haplotypes
LSC	88,033	210	125	85	0.00059	11
SSC	18,825	74	48	26	0.00089	11
IR	26,251	7	5	2	0.00006	7
Whole plastomes	159,360	298	183	115	0.00045	11

**Fig. 4:**
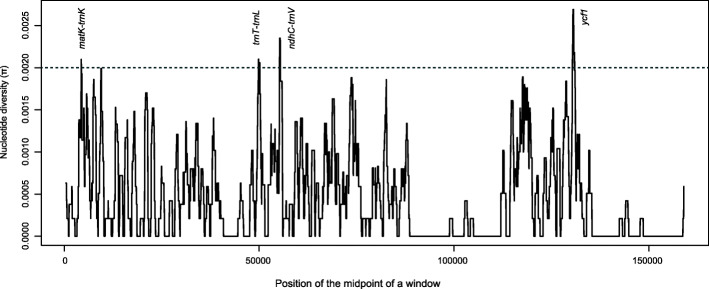
Nucleotide diversity (π) in the *Distylium* plastomes using sliding window method. The four mutation hotspot regions (π > 0.002) were annotated. π values were calculated in 800 bp sliding windows with 100 bp steps

The π value ranged from 0 to 0.0027 in an 800-bp sliding window size. In total, four peaks with π values > 0.002 were identified in the chloroplast genome (Fig. 4). Those regions included *matK-trnK*, *ndhC-trnV*, *ycf1*, and *trnT-trnL*. Three intergenic regions (*matK-trnK*, *ndhC-trnV*, and *trnT-trnL*) were located in the LSC region, and the *ycf1* coding region was in the SSC region. The primers were designed for the four variable markers (Table S3) and tested the effective for amplification (Figure S2).

**Fig. 5:**
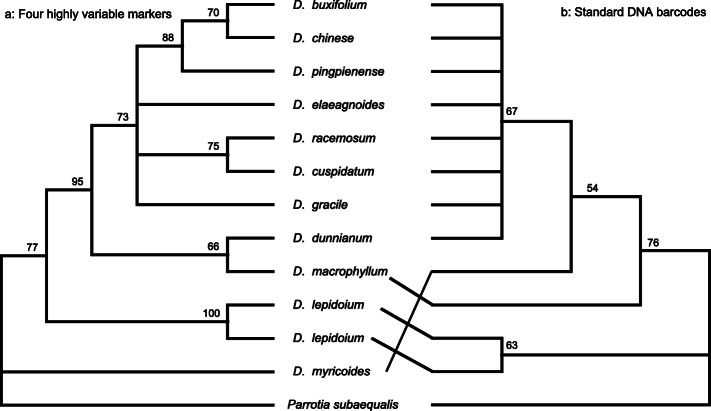
ML tree for *Distylium* using combine three universal plant DNA barcodes and four highly variable regions combinations

We tested the variability in the hypervariable markers by comparing with the three universal DNA barcodes (*matK*, *rbcL*, and *trnH-psbA*). The variable information is shown in Table 4. The intergenic spacer marker *trnH-psbA* was 367 bp, including two variable sites and no parsimony informative sites. The *rbcL* and *matK* genes were 1428 bp with three variable and three informative sites, and 1515 bp with only one variable and no informative sites, respectively. Combining the three universal markers, the aligned length was 3310 bp, with six variable sites and three informative sites. The mean distance was 0.00045. The species identification analyses showed that the universal DNA barcodes had less discriminatory power; there were only four haplotypes when combining the three markers, and the ML tree had lower resolution and most of the samples were not distinguished (Table 4 and Fig. 5).

**Table 4:** Variability of the four highly mutation hotspot regions and the universal chloroplast DNA barcodes in *Distylium*

Markers	Length (bp)	Polymorphic sites	Parsimony information sites	Mean distance	Nucleotide diversity	Number of haplotype
*matK-trnK*	827	8	3	0.00228	0.00227	7
*trnT-trnL*	1170	9	4	0.00184	0.00173	7
*ndhC-trnV*	961	6	4	0.00197	0.00198	7
*ycf1*	2306	20	5	0.00179	0.00179	9
Combination four variable markers	5264	43	16	0.00191	0.00197	11
*trnH-psbA*	367	2	0	0.00084	0.00084	2
*matK*	1515	1	0	0.00010	0.00010	2
*rbcL*	1428	3	3	0.00072	0.00072	3
Combination three universal markers	3310	6	3	0.00045	0.00045	4

The four hypervariable markers ranged from 827 bp (*matK-trnK*) to 2306 bp (*ycf1*) in length. The *ycf1* gene had the greatest number of variable sites (20 sites) followed by *trnT-trnL* (9 sites), *matK-trnK* (8 sites), and *ndhC-trrnV* had the fewest (6 sites). Combining the four hypervariable markers, there were 43 variable sites and 16 parsimony informative sites that produced the most current identification (Table 4). The identified hypervariable markers had higher resolution compared with the tree universal markers, based on the ML tree (Fig. 5). We also amplified and sequenced these four regions of two samples and used the tree-based methods to test their discrimination power. The results showed the two samples had successful identification (Figure S3).

### Phylogenetic inference

**Fig. 6:**
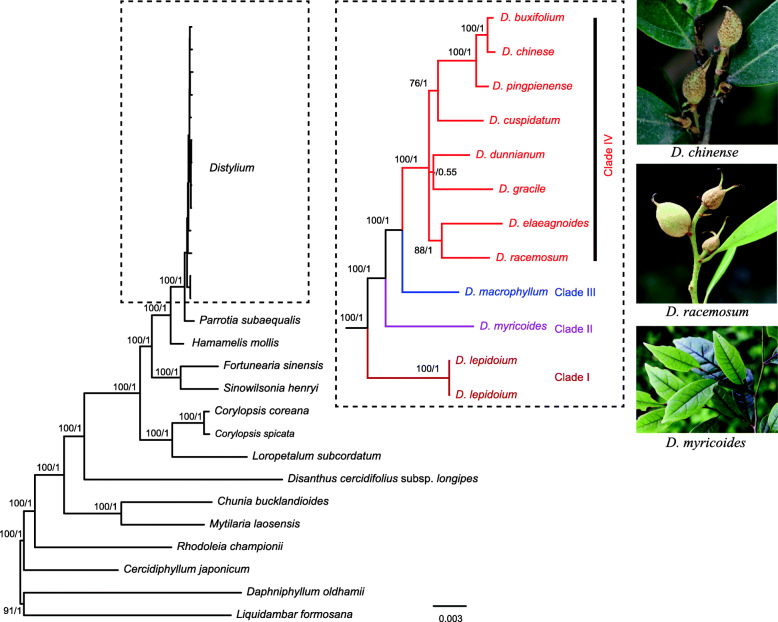
Phylogenetic reconstruction of Hamamelidaceae from Maximum likelihood (ML) and Bayesian inference (BI) methods based on the plastome dataset. The ML tree is shown. Number of the branches represent ML bootstrap support value (BP) /Bayesian posterior probability (PP). The photos were taken by ZhiXiang Zhang

Using the complete chloroplast genome sequences, we inferred the phylogenetic relationships among the 24 Hamamelidaceae samples. The best-fit model GTR + G from ModelFinder was used for ML and BI analyses. The topology of the ML and BI trees was nearly identical (Fig. 6). All *Distylium* species formed a monophyletic clade that was sister to *Parrotia* within Fothergilleae*. Distylium* had a short branch on the phylogenetic tree, indicating low divergence among *Distylium* species. Four clades were reconstructed in *Distylium* with a 100% bootstrap value. Clade I included the basal species *D. lepidoium*. Clade II included only *D. myricoides*. Clade III included only *D. macrophyllum*. Clade IV included the most advanced eight species, i.e., *D. buxifolium*, *D. chinense*, *D. pingienense*, *D. cuspidatum*, *D. dunnianum*, *D. gracile*, *D. elaeagoides*, and *D. racemosum* (Fig. 6).

### Estimate of divergence time

**Fig. 7:**
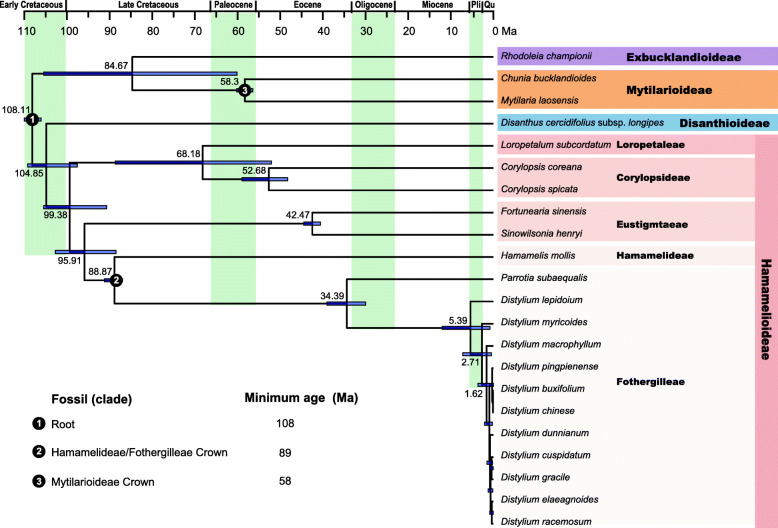
Divergence times of Hamamelidaceae obtained from BEAST analysis based on the complete plastome sequences. Mean divergence time of the nodes were shown next to the nodes while the blue bars correspond to the 95% highest posterior density (HPD). Black circles indicate the three calibration points

Divergence time estimates suggested that Hamamelioideae diverged from Hamamelidaceae about 99.38 Ma (95% HPD: 90.71–105.44 Ma) during the Cenomanian of the Upper Cretaceous (Fig. 7). The stem note of Fothergilleae was dated to 88.87 Ma (95% HPD: 97–91.18 Ma). The stem date for *Distylium* was estimated to be 34.39 Ma (95% HPD: 29.99–39.03 Ma) in the Oligocene and the *Distylium* crown date was 5.39 Ma (95%HPD: 0.82–12.3 Ma) in the Pliocene. Diversification within this genus occurred over a short time period of approximately 1 Ma.

## Discussion

The genera *Distyliopsis*, *Distylium*, *Fothergilla*, *Parrotia*, *Parrotiopsis*, *Shaniodendron*, and *Sycopsis* occur in the tribe Fothergilleae of the subfamily Hamamedoideae [9]. According to the phylogenetic relationships based on the several chloroplast and nuclear ITS genes [6, 8], *Distylium* is sister to *Distyliopsis* [9]. This is the first use of molecular data to infer the *Distylium* phylogeny. The *Distylium* genus formed a well-defined monophyletic group according to the chloroplast genome data (Fig. 6). Moreover, the phylogenetic tree possessed a series of short internodes within *Distylium* and most species diversified < 1 Ma (Fig. 7), suggesting that this genus has undergone rapid radiation. *D. lepidoium* was at the base of the genus. This species was first described in 1918 and is endemic to the Ogasawara Islands [4]. *D. myricoides* formed a monotypic clade and is distributed in eastern and southeastern China. According to the morphological characteristics, *D. myricoides* resembles *D. buxijolium* most closely, from which it may be distinguished by its larger leaves [5]. However, this relationship was not supported by the present study. *D. buxijolium* and *D. chinense* were sister species and formed a group supported by morphological characteristics [5]. In this study, the chloroplast genome data provided information to infer the phylogeny of *Distylium.* However, due to rapid radiation, sampling of additional individuals from each species and extending more nuclear genes would provide additional evidence of the evolutionary history of *Distylium.*

Most *Distylium* species are rare and endangered; thus, the development of rapid and easily accessible species identification methods is essential. The variations in the morphological characteristics between species were continuous and uninterrupted. Therefore, it was difficult to distinguish species using morphological characteristics. DNA barcoding offers an opportunity to identify *Distylium* species. *RbcL* and *matK* are the two core DNA barcodes in plants. However, many studies have shown that these two markers have lower species identification power [16, 17]. Our study also showed that *rbcL* and *matK* or a combination of the two markers failed to discriminate *Distylium* species (Fig. 5), explaining the low resolution in previous studies and highlighting the importance of developing highly divergent markers.

Some studies have indicated that mutations are not random and are clustered as “mutation hotspots” or “highly variable regions” [10, 16, 18]. In this study, we compared the whole chloroplast genomes and identified the mutation hotspots in *Distylium* (Fig. 4)*.* Four variable loci (*matK-trnK*, *ndhC-trnV*, *ycf1*, and *trnT-trnL*) were discovered. *TrnT-trnL* has been frequently used in plant phylogeny [19]. *MatK-trnK* and *ycf1* are considered divergence hotspots in angiosperms based on our previous research [16]. *NdhC-trnV* has been less used in plant phylogeny and species identification and is prone to have large indels [20]. The coding region of the *ycf1* locus is the most divergent marker in most groups, and has been suggested as the main plant DNA barcode [17]. *MatK-trnK* is located in the LSC region, and this locus is used less frequently in evolutionary biology. Some lineages have the ploy T structure [21]. Therefore, the lineage-specific, highly variable markers developed in this study will facilitate further phylogenetic reconstruction and DNA barcoding of rare and endangered *Distylium* species.

## Conclusions

In this study, we report 10 newly sequenced chloroplast genomes of *Distylium* species. The overall genomic structure, including the gene number and gene order, was well-conserved. The phylogeny and divergence time analyses based on the plastome sequences showed that *Distylium* was a rapidly radiating group and most speciation events occurred < 1 Ma. A comparison of sequence divergence across the *Distylium* plastomes revealed that *matK-trnK*, *ndhC-trnV*, *ycf1*, and *trnT-trnL* were mutation hotspot regions. Overall, our study demonstrated that plastome sequences can be used to improve phylogenetic resolution and species discrimination. Extended sampling and additional nuclear markers are absolutely necessary in further studies.

## Methods

### Plant material and DNA extraction

A total of 12 individual samples representing 11 *Distylium* species were sampled from the Plant DNA Bank of China at the Institute of Botany, Chinese Academy of Sciences. All samples were identified based on morphological characters. The details of the plant samples are presented in Table 5. Total genomic DNA was extracted from the leaf tissues of herbarium specimens of this genus following the modified CTAB DNA extraction protocol [22].

**Table 5:** Sampling information for the 12 *Distylium* samples

Species	Plant DNA bank of China	Collection locality
*Distylium dunnianum*	ENC850210	Rouan, Guangxi, China
*Distylium myricoides*	ENC850213	Jinggangshan, Jiangxi, China
*Distylium macrophyllum*	ENC850214	Rongshui Guangxi, China
*Distylium chinese*	ENC850215	Ruanling, Hunan, China
*Distylium racemosum*	ENC850217	Wuhu, Anhui, China
*Distylium pingpienense*	ENC850218	Napo, Guangxi, China
*Distylium buxifolium*	ENC850220	Shibing, Guizhou, China
*Distylium gracile*	ENC850222	Yilan, Taiwan, China
*Distylium cuspidatum*	ENC850224	Funing, Yunnan, China
*Distylium lepidoium*	ENC850418	Japan
*Distylium lepidoium*	ENC850420	Japan
*Distylium elaeagnoides*	ENC850421	Jianghua, Hunan, China

### Sequence, chloroplast genome assembly, and annotation

The total DNA was fragmented ultrasonically to construct350-bp insert libraries according to the manufacturer’s instructions, which was then used for sequencing. Paired-end sequencing was performed on an Illumina HiSeq X-ten at Novogene (Tianjin, China), yielding approximately 4 Gb of high-quality 150-bp paired-end reads per sample.

The raw reads obtained from Novogene were filtered using Trimmomatic 0.39 [23] with the following parameters: LEADING = 20, TRAILING = 20, SLIDING WINDOW = 4:15, MIN LEN = 36, and AVG QUAL = 20. High-quality reads were assembled de novo using the SPAdes 3.6.1 program [24]. The chloroplast genome sequence contigs were selected from the initial assembled reads in SPAdes by performing a BLAST search using several related Hamamelidaceae chloroplast genome sequences as references. The chloroplast genome sequence contigs were further assembled using Sequencher 5.4.5. All plastid assemblies were annotated in Plann [25] using *D. macrophyllum* (GenBank Accession number: MN729500) as the reference, and missing or incorrect genes were checked in Sequin. A circular diagram for the chloroplast genome was generated using OGDRAW [26]. All chloroplast genomes assembled in this study have been deposited in GenBank under accession numbers of MW248109 - MW248120.

### Microstructural mutation events

The Perl script microsatellite identification tool (MISA, http://pgrc.ipk-gatersleben.de/misa/misa.html) was used to identify the microsatellite regions of the chloroplast genome with the parameters set to 10 (repeat units ≥10) for mononucleotide simple sequence repeats (SSRs), 6 (repeat units ≥6) for dinucleotides, 5 (repeat units ≥5) for trinucleotides, 4 (repeat units ≥4) for tetranucleotides, and 3 (repeat units ≥3) for pentanucleotides and hexanucleotides.

The chloroplast genomes sequences were aligned using MAFFT [27] followed by manually examination and adjustment. Based on the aligned sequence matrix, the indels were manually checked and divided into categories of repeat indels and normal indels, according to Dong et al. [15]. *D. dunnianum* was used as the reference to determine the size and position of the indel events.

### Sequence divergence analysis

The mVISTA program was used to compare the variability of *Distylium* chloroplast genome using the Shuffle-LAGAN mode [28]. Single nucleotide substitutions and the genetic p-distances were calculated using MEGA 7.0 [29] based on the aligned chloroplast genome sequences. To assess sequence divergence andexplore highly variable chloroplast markers, nucleotide diversity (π) was calculated by sliding window analysis using DnaSP v6 [30] with a widow size of 800 bp and a step size of 100 bp. The primers for amplifying the highly variable regions were designed using FastPCR [31]. The PCR amplifications were performed following Dong et al. [32].

Nucleotide diversity and the number of haplotypes were used to assess marker variability for all barcodes (hype-variable markers and the universal plant DNA barcodes, *rbcL*, *matK*, and *trnH-psbA*). The tree-based method was utilized to evaluate discrimination power. A maximum-likelihood (ML) tree was prepared in IQ-TREE2 using the GTR model [33].

### Phylogenetic analyses

To elucidate the phylogenetic positions of *Distylium* within Hamamelidaceae and the interspecific phylogenetic relationships within *Distylium*, multiple alignments were performed using the whole chloroplast genome of 24 Hamamelidaceae samples representing 11 genera, including *Cercidiphyllum japonicum, Daphniphyllum oldhamii*, and *Liquidambar formosana* as outgroups. The Hamamelidaceae chloroplast genomes were aligned using MAFFT, and ambiguous alignment regions were trimmed with Gblocks 0.91b [34]. The maximum-likelihood (ML) analysis was run with RAxML-NG [35] with the best-fit model from ModelFinder [36]. Branch support was assessed by fast bootstrap methodology using non-parametric bootstrapping and 500 ML pseudo-replicates.

Mrbayes v3.2 [37] was used to infer the Bayesian inference (BI) tree. The BI analysis was run for 20 million generations, in which a tree was sampled every 1000 generations. Two independent Markov Chain Monte Carlo (MCMC) analyses were performed and each chain started with a random tree. The first 25% of the sampled trees was discarded as burn-in, while the remaining trees were constructed in a majority-rule consensus tree to estimate posterior probabilities.

### Molecular clock dating

We used BEAST v2.5.1 [38] to estimate the divergence times of Hamamelidaceae using three priors based on the complete plastome sequences. Based on the average value obtained by Xiang et al. [9] in a calibrated analysis, three priors were used: (i) the average age of the most recent common ancestor (TMRCA) of Hamamelidaceae (the root of the tree) was 108 Ma; (ii) the crown age of Hamamelideae/Fothergilleae was 89 Ma; and (iii) the crown age of Mytilarioideae was 58.3 Ma. Each secondary prior was placed under a normal distribution with a standard deviation of 1.

The GTR nucleotide substitution model and the prior tree Yule model were selected with the uncorrelated lognormal distribution relaxed molecular clock model. The MCMC run had a chain length of 400,000,000 generations with sampling every 10,000 generations. The stationary phase was examined through Tracer 1.6 [39] to evaluate convergence and to ensure sufficient and effective sample size for all parameters surpassing 200. A burn-in of 10% generations was discarded, and TreeAnnotator v2.4.7 was used to produce a maximum clade credibility tree.

## Supplementary Information

**Additional file 1: Table S1.** List of genes found in the *Distylium* chloroplast genome.

**Additional file 2: Table S2.** More detail of SSRs in *Distylium* species.

**Additional file 3: Table S3.** The primers used for amplification the variable markers.

**Additional file 4: Figure S1.** Visualization of the alignment of chloroplast genome sequences of *Distylium*. VISTA-based similarity graphical information illustrating the sequence identity of *Distylium* with reference *D. chinese* chloroplast genomes. The Y-scale axis represents the percent identity within 50–100%.

**Additional file 5: Figure S2.** Gel profiles of fragments amplified from two species using four pairs of primers.

**Additional file 6: Figure S3.** ML tree for *Distylium* using four highly variable regions combinations.

## Data Availability

The chloroplast genome of *Distylium* assembled in this study have been deposited in the National Center for Biotechnology and Information (NCBI) under the following accession as summarized in Table 1. The other sequences used in this study were downloaded from the NCBI.

## Abbreviations

BI: Bayesian Inference
bp: Base pairs
Gb: Gigabases
LSC: Long single copy
Ma: Million years ago
MCMC: Markov chain Monte Carlo
ML: Maximum likelihood
NCBI: National Center for Biotechnology Information
NGS: Next generation sequencing
π: Nucleotide diversity
rRNA: Ribosomal RNA
SSC: Short single copy
SSR: Simple sequence repeat
tRNA: Transfer RNA
