# Supplementary material for: Chloroplast phylogenomic insights into the evolution of Distylium (Hamamelidaceae)
Source: BMC Genomics. 2021 Apr 22;22:293. doi: 10.1186/s12864-021-07590-6 (PMC8060999; doi:10.1186/s12864-021-07590-6)
Supplement: Supplementary file 1 — Additional file 1: Table S1. List of genes found in the Distylium chloroplast genome. [file 12864_2021_7590_MOESM1_ESM.docx]

**Table S1**. List of genes found in the *Distylium* chloroplast genome. Intron-containing genes are marked by asterisks (*).

| **Category for genes** | **Group of gene** | **Name of gene** |
| --- | --- | --- |
| Photosynthesis related genes | Rubisco | *rbcL* |
|  | Photosystem Ⅰ | *psaA,psaB,psaC,psaI,psaJ* |
|  | Assembly/stability of photosystem Ⅰ | **ycf3,ycf4* |
|  | Photosystem Ⅱ | *psbA,psbB,psbC,psbD,psbE,psbF,psbH,psbI,psbJ,psbK,psbL,psbM,psbN,psbT,psbZ* |
|  | ATP synthase | *atpA, atpB, atpE, *atpF, atpH, atpI* |
|  | cytochrome b/f compelx | *petA, *petB, *petD, petG, petL, petN* |
|  | cytochrome c synthesis | *ccsA* |
|  | NADPH dehydrogenase | **ndhA, *ndhB, ndhC, ndhD, ndhE, ndhF ,ndhG, ndhH, ndhI, ndhJ, ndhK* |
| Transcription and translation related genes | transcription | *rpoA, rpoB, *rpoC1, rpoC2* |
|  | ribosomal proteins | *rps2, rps3, rps4, rps7, rps8, rps11, *rps12, rps14,rps15, *rps16, rps18, rps19,*rpl2, rpl14, *rpl16, rpl20, rpl22, rpl23, rpl32, rpl33,rpl36* |
|  | translation initiation factor | *infA* |
| RNA genes | ribosomal RNA | *rrn5, rrn4.5, rrn16, rrn23* |
|  | transfer RNA | **trnA*_UGC_*, trnCGCA, trnDGUC, trnEUUC, trnFGAA,trnGGCC, *trnGUCC, trnHGUG, trnICAU, *trnIGAU,*trnKUUU, trnLCAA, *trnLUAA, trnLUAG, trnfMCAUI,trnMCAU, trnNGUU, trnPUGG, trnQUUG,trnRACG, trnRUCU, trnSGCU, trnSGGA, trnSUGA, trnTGGU,trnTUGU, trnVGAC, *trnVUAC, trnWCCA, trnYGUA* |
| Other genes | RNA processing | *matK* |
|  | carbon metabolism | *cemA* |
|  | fatty acid synthesis | *accD* |
|  | proteolysis | **clpP* |
| Genes of unknown function | conserved reading frames | *ycf1, ycf2,* |
