# Supplementary figures and images for: Chloroplast phylogenomic insights into the evolution of Distylium (Hamamelidaceae)
Source: BMC Genomics. 2021 Apr 22;22:293. doi: 10.1186/s12864-021-07590-6 (PMC8060999; doi:10.1186/s12864-021-07590-6)

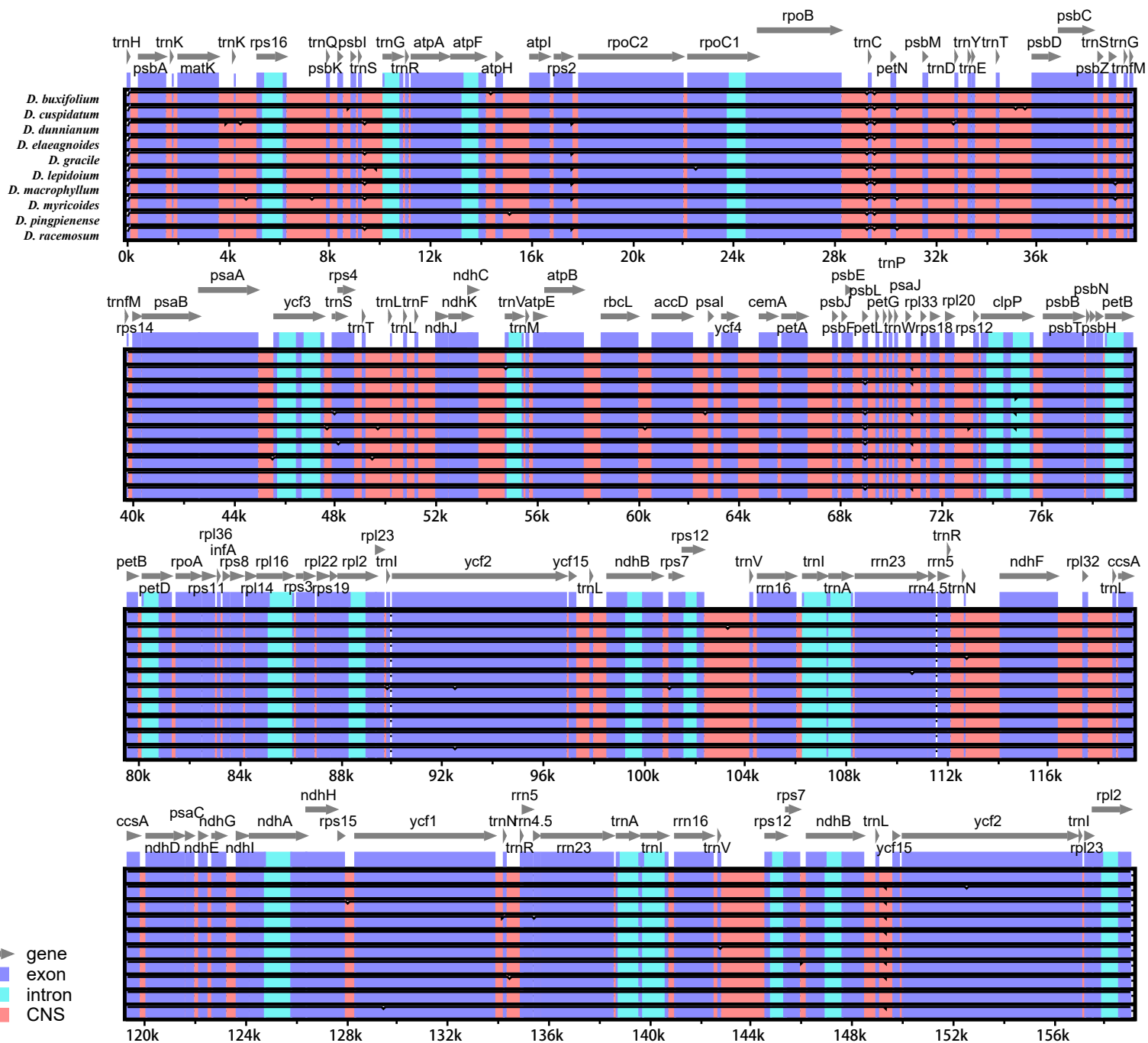

Supplement: Supplementary file 4 — Additional file 4: Figure S1. Visualization of the alignment of chloroplast genome sequences of Distylium. VISTA-based similarity graphical information illustrating the sequence identity of Distylium with reference D. chinese chloroplast genomes. The Y-scale axis represents the percent identity within 50–100%. [file 12864_2021_7590_MOESM4_ESM.pdf]

POC506454

POC529237

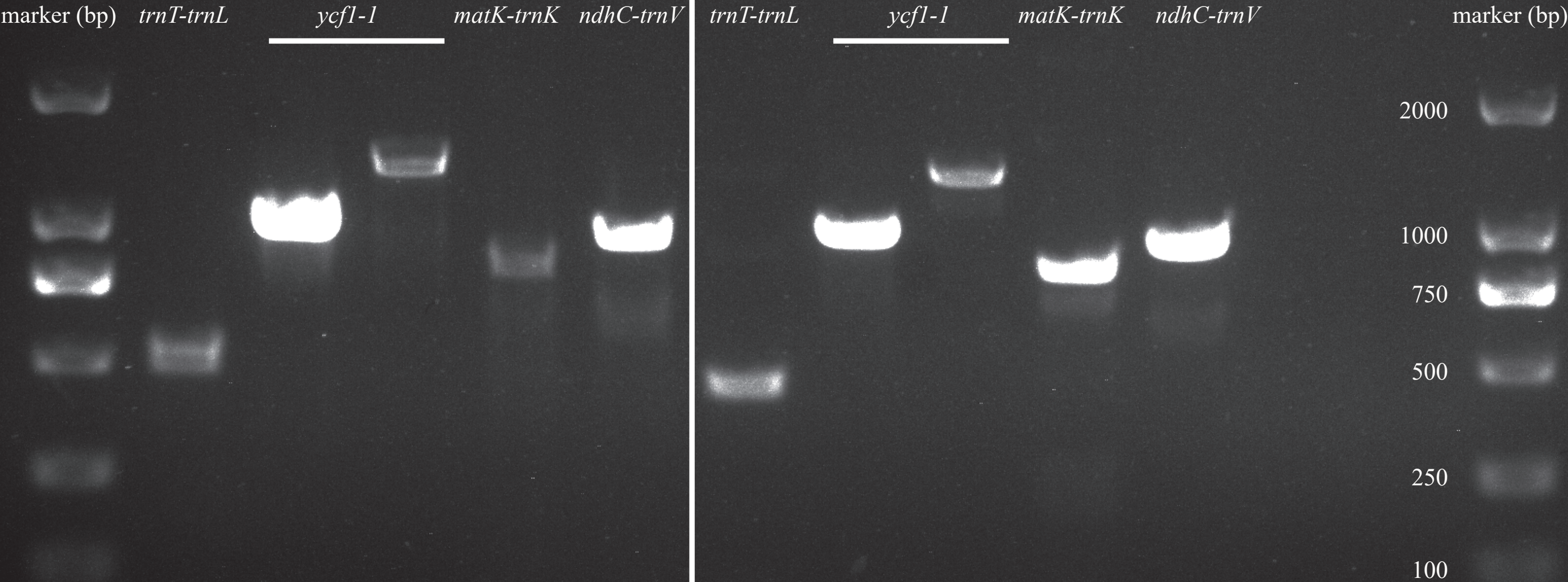

Supplement: Supplementary file 5 — Additional file 5: Figure S2. Gel profiles of fragments amplified from two species using four pairs of primers. [file 12864_2021_7590_MOESM5_ESM.pdf]

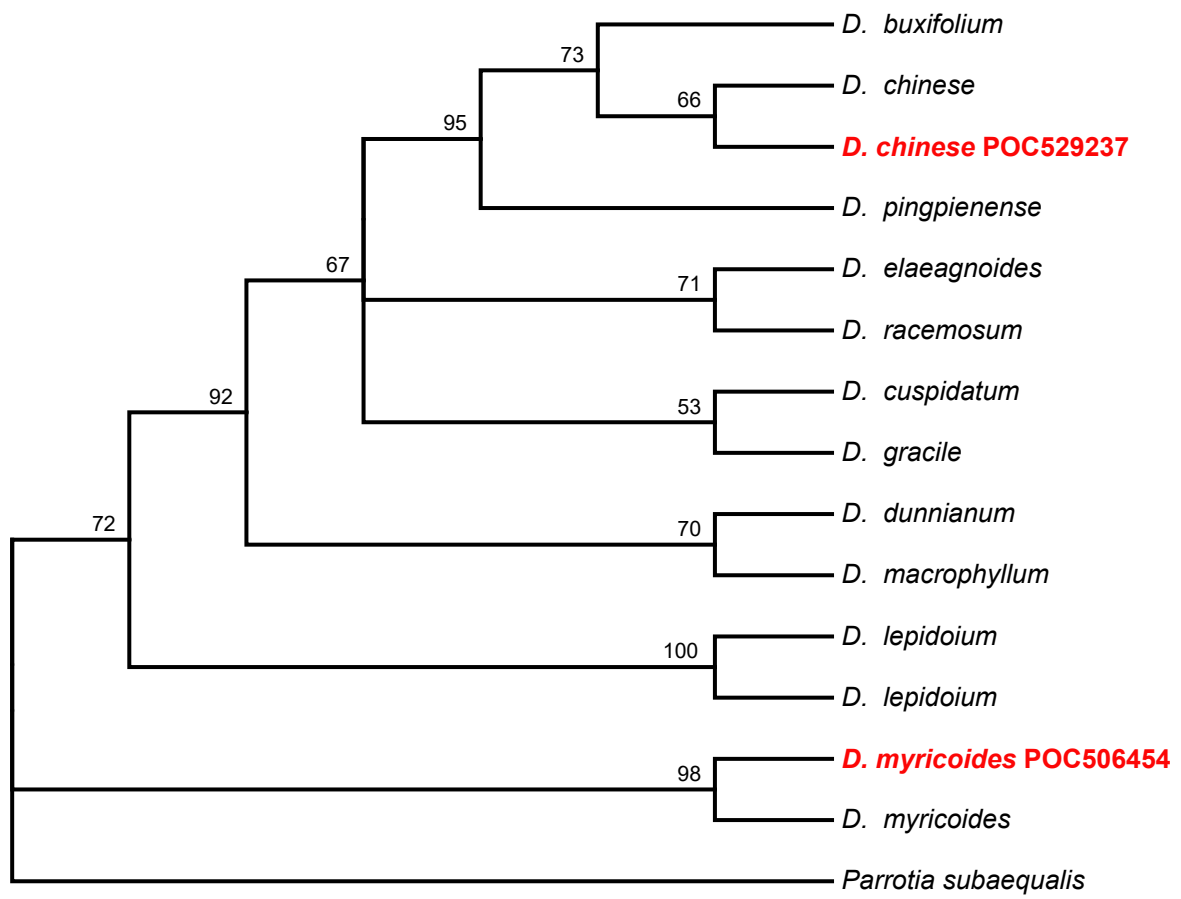

Supplement: Supplementary file 6 — Additional file 6: Figure S3. ML tree for Distylium using four highly variable regions combinations. [file 12864_2021_7590_MOESM6_ESM.pdf]
